# Basal Plane Activation of SnS_2_ Thin‐Film by Fluorine Doping for Selective Solar‐Driven CO_2_ Reduction With Enhanced Quantum Efficiency

**DOI:** 10.1002/advs.202522924

**Published:** 2026-01-20

**Authors:** Tadios Tesfaye Mamo, Mohammad Qorbani, Adane Gebresilassie Hailemariam, Putikam Raghunath, Che‐Men Chu, Wen‐Hsin Yuan, Amr Sabbah, Yen‐Yu Wang, Shuo‐Yun Chang, Ming‐Chang Lin, Wei‐Yen Woon, Yu‐Jung Lu, Heng‐Liang Wu, Ken‐Tsung Wong, Kuei‐Hsien Chen, Li‐Chyong Chen

**Affiliations:** ^1^ Center for Condensed Matter Sciences National Taiwan University Taipei Taiwan; ^2^ Center of Atomic Initiative For New Materials National Taiwan University Taipei Taiwan; ^3^ Department of Chemistry National Taiwan University Taipei Taiwan; ^4^ Institute of Atomic and Molecular Sciences Academia Sinica Taipei Taiwan; ^5^ Undergraduate Program of Electro‐Optical Engineering National Taiwan Normal University Taipei Taiwan; ^6^ Institute of Electro‐Optical Engineering National Taiwan Normal University Taipei Taiwan; ^7^ Department of Chemistry College of Natural Science Dire Dawa University Dire Dawa Ethiopia; ^8^ Department of Applied Chemistry National Yang Ming Chiao Tung University Hsinchu Taiwan; ^9^ Department of Physics National Central University Jungli Taiwan; ^10^ Tabbin Institute For Metallurgical Studies Helwan Cairo Egypt; ^11^ Department of Physics National Taiwan University Taipei Taiwan; ^12^ Research Center For Applied Sciences Academia Sinica Taipei Taiwan; ^13^ Department of Chemistry National Tsing‐Hua University Hsinchu Taiwan; ^14^ National Synchrotron Radiation Research Center Hsinchu Taiwan

**Keywords:** in situ FTIR, in situ NAP‐XPS, ion implantation, photocatalysis, reaction pathway, thin film

## Abstract

Photocatalytic conversion of CO_2_ into value‐added fuels offers a viable approach to combat climate change and address global energy demands. Here, we present a fluorine‐doped SnS_2_ thin film with sulfur vacancy (i.e., S_V_‐SnS_2_:F), prepared via thermal evaporation, post‐sulfurization, and fluorine ion‐implantation. Substitution of sulfur with fluorine and sulfur vacancy formation changes the product selectivity from CH_4_ to CO with about 40‐fold enhanced yield and boosted internal quantum efficiency (***IQE***) of 0.52%. Transient absorption, in situ near‐ambient pressure X‐ray photoelectron, and in situ Fourier transform infrared spectroscopies, along with first‐principles density functional theory calculations, suggest that nearest‐neighbor Sn to F serves as an active site and stabilizes the *COOH intermediate. Our findings shed light on how F doping activates the nearby elements and its crucial role in intermediate stabilization toward selectivity change in a heterogeneous photocatalysis process.

## Graphical abstract

The synergistic effect of fluorine doping and sulfur vacancies boosts the activity of the active sites without active‐site modulation, leading to enhanced CO_2_ photoreduction efficiency and a selectivity switch from CH_4_ to CO on tin disulfide continuous thin films.

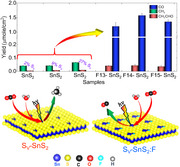

## 1 Introduction

Photocatalytic conversion of carbon dioxide (CO_2_) into other invaluable products such as CO and CH_4_ is potentially an important route toward a net‐zero‐emission society [1, 2, 3, 4, 5, 6, 7, 8]. Despite its dual benefits, i.e., mitigating greenhouse gas emissions and generating renewable fuels, finding an efficient photocatalyst is still challenging. In this regard, numerous efforts have been made to the rational design of photocatalysts with enhanced light‐harvesting capabilities, improved charge separation and transport properties, abundant active sites, and increased photostability [2, 9, 10, 11]. Among the wide variety of heterogeneous photocatalysts, tin disulfide (SnS_2_) has gained considerable attention due to its non‐toxicity, cost‐effectiveness, suitable bandgap (∼1.9–2.5 eV), electronic band alignments for redox reaction, and excellent chemical stability. However, it suffers from a lack of active sites and rapid recombination of charge carriers [12, 13]. So far, to enhance its photocatalytic (PC) activity for CO_2_ reduction, different strategies such as doping [12, 14], sulfur vacancies (S_V_) engineering [15], and a S_V_–dopant pairing have been employed [13]. These studies highlight the importance of active site modulation, which plays a pivotal role in enhancing photocatalytic performance. However, boosting the activity of an active site without modulating it, while concurrently attaining better selectivity and precise product tuning, continues to pose a significant challenge in the field. In this regard, the type and concentration of dopants are crucial, as they fundamentally modify the electronic structure, charge distribution, and local lattice arrangement, thereby providing an effective strategy to address this challenge.

Among various dopants, fluorine (F) has attracted significant attention owing to its low atomic mass, high electronegativity, abundance, and strong bonding capability with metals and non‐metals, making it a powerful strategy for modulating the electronic structure and enhancing catalytic activity in photocatalytic and electrocatalytic systems. For instance, F doping largely modifies the optoelectronic properties of 2D electronic devices [16], improves visible‐light absorption, promotes charge separation, increases free‐electron density, and enhances surface hydrophilicity, resulting in more efficient generation of photocatalytic reactive oxygen species (ROS) in metal oxides such as TiO_2_ and ZnO [17, 18]. F incorporation also helps weaken the ^*^OH (asterisks indicate catalytically active sites) binding energy, stabilizes the ^*^OOH intermediate, and constructs a local superhydrophobic interface that easily enhances the diffusion of H_2_O and the mass transport in oxygen reduction reactions (ORR) [19]. Moreover, incorporation of F in Cu particles creates grain boundaries, and this change in the microenvironment stabilizes the CO intermediate, favors the subsequent C─C coupling, and boosts C_2_^+^ product generation while suppressing the competitive hydrogen evolution reaction (HER) [20]. Similarly, F doping activates nearby carbon atoms in the carbon matrix by creating asymmetric charge distributions and enhanced polarization, resulting in increased CO product selectivity through stabilization of the key reaction intermediate ^*^COOH in electrocatalytic CO_2_ reduction [21]. Yuan et al., further demonstrated that a polarized carbon atom serves as an effective site for proton adsorption by N_2_ molecules, thereby enhancing nitrogen reduction reaction (N_2_RR) performance [22]. Additionally, F doping alters the electronic configuration of the central Ni–N_4_ sites, favouring ^*^COOH formation by lowering the energy barrier of CO_2_ activation [23]. Hence, introducing F dopants into a lattice in a precise and controllable manner is crucial for tailoring material properties. Among various doping strategies, ion implantation is particularly advantageous because it offers precise control over dopant species, implantation energy, and dose. This enables uniform dopant distribution laterally and a controllable profile in depth, with excellent reproducibility and scalability [24, 25, 26].

Herein, we synthesize a F ion‐implanted SnS_2_ thin film by thermal evaporation, post‐sulfurization, and ion‐implantation processes. Due to the impingement of F ions into SnS_2_ at high kinetic energy, it leaves sulfur vacancies (S_V_). Further, Experimental and computational results unveil that F substitutes S (S_V_‐SnS_2_:F) and the nearest‐neighbor Sn to F serves as an active site for PC CO_2_ reduction. It results in a stabilization of rate‐determining intermediate ^*^COOH and gives a product tuning from CH_4_ (of 0.04 µmol cm^−2^ for pristine SnS_2_) to CO (of 1.57 µmol cm^−2^ for S_V_‐SnS_2_:F) with a selectivity of ∼97% and an internal quantum efficiency (*IQE*) of 0.52%, which is about 30 times larger than pristine SnS_2_. In addition, the enhanced transferred electron rate (*R_e_*) from 0.5 to 2.2 e^−1^ s^−1^ per active site (i.e., nearest‐neighbor Sn to F) for the optimal S_V_‐SnS_2_:F sample, compared with the pristine SnS_2,_ illustrates the role of F in boosting the activity of the nearby Sn site.

## 2 Results and Discussion

### 2.1 Film Morphology and Structural Properties

**FIGURE 1:**
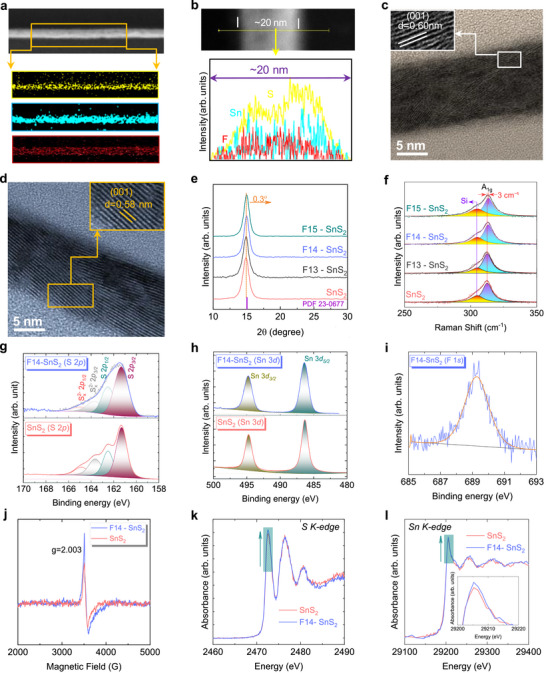
Thin film characterizations. (a,b) Cross‐sectional line scan and EDX elemental mapping images of Sn, S, and F elements in the F‐doped SnS_2_ film, respectively. (c,d) Cross‐sectional HRTEM images of SnS_2_ and F14‐SnS_2_, respectively. (e,f) XRD patterns and Raman spectra of SnS_2_ and F‐doped SnS_2_ thin films, respectively. (g,h) XPS spectra of Sn, S, and F, respectively. (j) The EPR spectra of the thin films. (k,l) XAS spectra of S, Sn k‐edge, respectively.

20‐nm F‐doped SnS_2_ continuous thin films were prepared using thermal evaporation and post sulfurization processes as described by Mamo et al. [13], followed by an ion implantation process to introduce varying amounts of F into the SnS_2_ (see Note S1). Cross‐sectional transmission electron microscopy (TEM) images, along with their corresponding energy‐dispersive X‐ray spectroscopy (EDX) elemental mapping and line scan, reveal a uniform distribution of Sn, S, and F elements (Figure 1a,b). As shown in Figure 1c,d, the high‐resolution transmission electron microscopy (HRTEM) images indicate a slight decrease in *d*‐spacing after F doping. Similarly, the X‐ray diffraction (XRD) peak for the (001) plane exhibits a 0.3° shift toward a higher diffraction angle (Figure 1e), indicating a shrinkage of the crystal structure after the implantation of F ions. As illustrated in Figure 1f, the A_1g_ out‐of‐plane Raman vibrational mode of SnS_2_ shows a blueshift of approximately 3 cm^−1^ due to the substitution of light F into the relatively heavier S, which is consistent with the harmonic oscillator model. It should also be noted that Si vibrational mode is shown at ∼303 cm^−1^, which comes from the substrate [2]. Additionally, our density functional theory (DFT) calculations (Figure S1 and Table S1) suggest that fluorine preferentially substitutes sulfur rather than tin, or occupies interstitial sites between the layers. In the following text, we will discuss the probability of substitution or interstitial configurations.

X‐ray photoelectron spectroscopy (XPS) was employed to analyze the oxidation states and stoichiometry of the thin films (see Figure 1g–i; Table S2). The spectra exhibited peaks at binding energies of 486.28 eV (Sn 3*d*_5/2_), 494.70 eV (Sn 3*d*_3/2_), 161.31 eV (S 2*p*_3/2_), and 162.51 eV (S 2*p*_1/2_), indicating that tin predominantly exists as Sn^4+^ and sulfur is in the S^2−^ and S_x_^2−^ oxidation states [27]. Following F doping, a peak emerged at 689.2 eV, corresponding to the F 1*s* signal [21, 28]. A relative S 2*p* peak intensity reduction was also observed after F implantation, suggesting the substitution of F into S sites as well as the formation of S_V_. Therefore, the (S+F)/Sn ratio decreased from 1.96 (with 2% S_V_) to 1.85 (with 7.5% S_V_), while the F/(Sn+S+F) ratio increased from 0 to 7.2% after ion implantation, hereafter denoted as F14‐SnS_2_.

Figure 1j reveals that F14‐SnS_2_ exhibits a stronger electron paramagnetic resonance (EPR) signal and peak broadening compared to SnS_2_ at g = 2.003, indicating a higher level of S_V_ [29]. The asymmetric EPR line shape in both SnS_2_ and F‐doped SnS_2_ arises from Dysonian resonance behavior caused by exchange coupling between defect‐related localized spins and delocalized charge carriers, with F doping further enhancing this interaction through increased carrier density and defect stabilization [30, 31, 32]. The Sn and S *K*‐edges of both SnS_2_ and F‐doped SnS_2_ thin films have been measured to examine their bulk electron transfer properties using X‐ray absorption near‐edge spectroscopy (XANES). As depicted in Figures 1k,l, the white line intensities for both Sn and S are enhanced in F‐doped SnS_2_. These changes confirm that the Sn and S atoms are losing electrons. Bader charge analysis (Figure S2) further supports this observation, showing that F atoms preferentially occupy sulfur sites, which is in good agreement with the abovementioned experiments.

### 2.2 Band Structure and Charge Transfer

**FIGURE 2:**
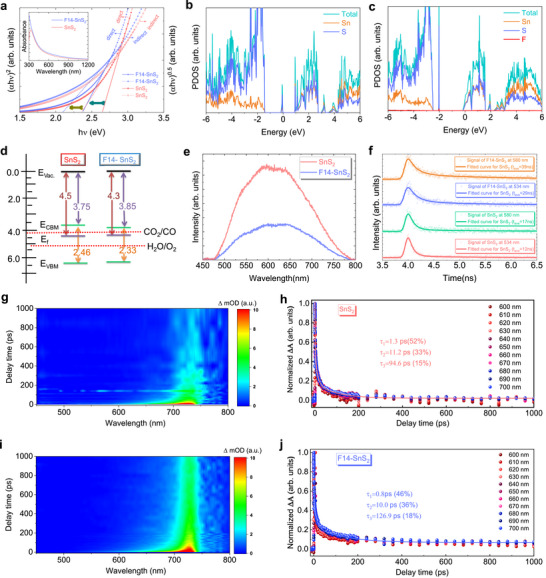
Charge transfer dynamics. (a) Tauc plot curves of the pristine and F‐doped SnS_2_ for both direct and indirect optical transitions. The inset shows the UV–visible spectra. (b,c) PDOS of S_V_‐SnS_2_ and S_V_‐SnS_2_:F, respectively. (d) Comparative band diagram of S_V_‐SnS_2_ and S_V_‐SnS_2_:F. (e) PL spectrum at room temperature. (f) TRPL data and fitted curves for pristine SnS_2_ and F14‐SnS_2_ films at 534 and 580 nm. (g,h) Color maps of FsTA spectra and decay kinetics at different probe wavelengths for SnS_2_, respectively. (i,j) Color maps of FsTA spectra and decay kinetics at different probe wavelengths for F14‐SnS_2_, respectively.

Following F incorporation, absorbance spectra and corresponding Tauc plots reveal a slight reduction in the optical bandgaps, along with enhanced light‐harvesting efficiency (Figure 2a) [33, 34]. As shown in Figure 2b, S_V_‐SnS_2_ exhibits a valence band maximum (VBM) dominated by S 3*p* states at approximately −1.43 eV, while the conduction band minimum (CBM) is primarily composed of Sn 5*p* orbitals located near +0.93 eV. The introduction of sulfur vacancies generates pronounced defect states within the bandgap at around −0.07 eV relative to the Fermi level, originating from coordinatively unsaturated Sn atoms adjacent to the vacancy. These localized mid‐gap states substantially narrow the effective bandgap and impart n‐type character; however, they may also serve as charge‐trapping and recombination centers.

In contrast, fluorine incorporation at the vacancy site (S_V_‐SnS_2_:F) markedly alters the electronic structure(Figure 2b,c; Figure S3). The vacancy‐induced mid‐gap states are largely suppressed and redistributed toward the band edges due to strong Sn–F orbital hybridization, indicating effective defect passivation. Quantitatively, the CBM shifts downward from +0.93 to −0.13 eV, while the VBM moves deeper to −2.48 eV. Residual defect states associated with S and Sn appear at approximately 0.45 eV above the valence‐band edge (−2.03 eV). Consequently, the Fermi level shifts closer to the CBM, accompanied by an increased density of states near EF. This enhanced electronic delocalization implies improved n‐type conductivity and more efficient electron transfer, providing a clear electronic origin for the superior catalytic performance of S_V_–SnS_2_:F. Furthermore, ultraviolet photoemission spectroscopy (UPS) and low‐energy inverse photoemission spectroscopy (LEIPS) measurements indicate that the work function decreases with the incorporation of F, resulting in a downward shift of the conduction band edge relative to the vacuum level (Figure 2d; Figure S4).

Photoluminescence (PL) spectroscopies, along with time‐resolved photoluminescence (TRPL) measurements, were carried out to probe charge‐transfer dynamics. The unmodified SnS_2_ sample shows a broad PL emission in the visible region, with average exciton lifetimes of 0.12 ns at 534 nm (and 0.17 ns at 580 nm). Conversely, the F‐SnS_2_ sample, due to substantial non‐radiative recombination of charge carriers, exhibits a significantly weaker PL signal, with longer average lifetimes of 0.29 ns at 534 nm (and 0.39 ns at 580 nm), as presented in Figure 2e,f. Moreover, the ultrafast transient absorption spectroscopy (TAS) further highlighted the role of F implantation in SnS_2_. The exciton average recombination time of optical transitions was determined by fitting the TAS signals. The color maps of SnS_2_ and F14‐SnS_2_ show a broad photo‐induced absorption, as shown in Figure 2g,i. We attribute this broadband absorption to the defect‐trapped charge carriers. As depicted in Figure 2h,j, the TAS traces of both SnS_2_ and F14‐SnS_2_ were well‐fitted with a triexponential function, resulting in three distinct decay lifetimes (τ_1_, τ_2_, and τ_3_). The shortest lifetime (∼1 ps) likely corresponds to hot carrier relaxation, while the second (10 ps) and third lifetimes (100 ps) may represent carriers trapped in shallow and deep defects, respectively [35, 36]. F14‐SnS_2_ had shorter decay lifetimes of τ_1_ = 0.8 ps and τ_2_ = 10 ps compared to SnS_2_ with decay lifetimes of τ_1_ = 1.3 ps and τ_2_ = 11.2 ps [37, 38, 39]. This means that excitons in the F14‐SnS_2_ sample migrate faster from the conduction band to the shallow trap states, allowing them to dissociate into free electrons more easily. Furthermore, the longer lifetime τ_3_ = 126.9 ps in F14‐SnS_2_ compared to τ_3_ = 94.6 ps in SnS_2_ indicates a prolonged process of electron capturing and accumulation by deep trap states, along with their recombination with valence band holes. These results reveal that implanting F into SnS_2_ efficiently suppresses deep trap states and direct recombination of photogenerated electrons, resulting in improved exciton dissociation and charge transfer efficiency.

### 2.3 Photocatalytic CO_2_ Reduction and Intermediates

**FIGURE 3:**
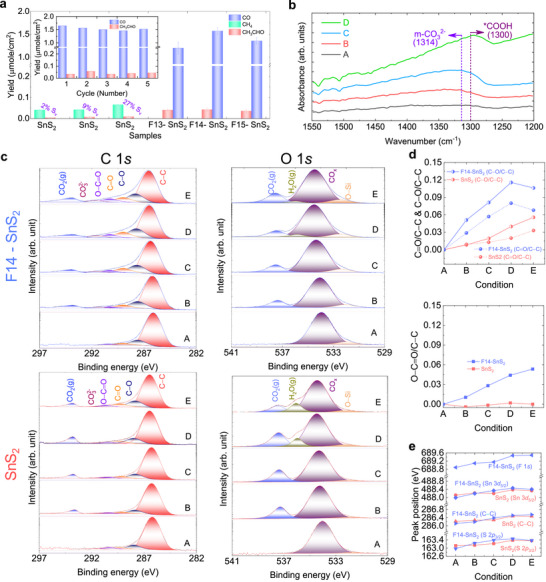
Photocatalytic CO_2_ reduction. (a) Blank‐corrected total CO yield for 4 h. Inset shows the stability test of the F14‐SnS_2_ sample for five cycles. (b) In situ FTIR spectra of F‐SnS_2_ thin film at different conditions: (A) Before purging humidified CO_2_; (B) after purging humidified CO_2_ (H_2_O+CO_2_) for 1hr without light irradiation; and in CO_2_ + H_2_O atmosphere with light illumination for (C) 2 h and (D) 4 h. (c) In situ NAP‐XPS spectra of C 1*s* and O 1*s* for pristine and F‐doped SnS_2_ at different conditions: (A) UHV; (B) In a CO_2_ atmosphere in the dark; (C) In a 1 mbar CO_2_ atmosphere under light illumination; (D) In a 1 mbar CO_2_ + 1 mbar H_2_O atmosphere in the dark; and (E) In a 1 mbar CO_2_ + 1 mbar H_2_O atmosphere under light illumination. (d) C─O/C─C, C═O/C─C, O─C═O/C─C peak ratios at different conditions calculated from the C 1*s* spectrum. (e) Sn 3*d*_5/2_, S 2*p*_3/2_, F 1*s*, C─C peak positions for the pristine and F‐doped SnS_2_ thin films at different conditions.

The PC experiments were carried out in a humidified CO_2_ atmosphere under visible light with water. As shown in Figure 3a, pristine SnS_2_ yields 0.04 µmol cm^−2^ of CH_4_ and a negligible amount of acetaldehyde. In contrast, we obtained CO as the main product for the optimized F‐doped SnS_2_ sample, as well as acetaldehyde as a minor product. The CO productivity reaches 1.57 µmol cm^−2^, with a selectivity of approximately 97% and an *IQE* of ∼0.52%. It should be noted that the overall apparent quantum efficiency (*AQE*) shows about 30 times enhancement in the PC activity from ∼0.002 to ∼0.06% after F incorporation (see Table S3 for the definitions of *IQE* and *AQE*). Compared with other reported systems, this sample shows among the highest CO selectivity and productivity in both powder and thin‐film photocatalysts (Table S4). Additionally, we calculated the transferred electron rate (*R_e_*) [2], where pristine SnS_2_ exhibited *R_e_* = 0.5 e^−^ s^−1^ per S_V_. Remarkably, the F‐doped sample showed over a fourfold increase, with *R_e_* = 2.2 e^−^ s^−1^ per S_V_. It implies the impact of F in the activation of Sn connected to F for efficient charge transfer to the adsorbed reactants, beyond changing the reaction pathway. Furthermore, as shown in the inset of Figure 3a, the optimized F‐doped SnS_2_ sample maintained stable performance over five consecutive cycles. XRD peaks and Raman analyses after stability test (Figures S5 and S6) confirmed that its crystal structure remained intact. To further demonstrate the synergistic effect between F doping and sulfur vacancy (S_V_) pairing, we conducted a series of photocatalytic (PC) experiments on pristine SnS_2_ with a larger amount of S_V_ of 9 % and 27 % [13]. The results show that increasing the S_V_ density leads to a slight enhancement in CH_4_ production, but still no CO product is observed. This observation indicates that although S_V_ can marginally promote CH_4_ formation, S_V_ without pairing with F doping is not an efficient active site for CO_2_ activation and selective reduction. To identify the source of the detected CO, a ^13^CO_2_ isotope tracer experiment was carried out under identical reaction conditions. Figure S7a, shows a prominent signal at *m*/*z* = 29, which corresponds to ^13^CO, indicating that CO_2_ is the source of the observed CO product. The peak at *m*/*z* = 28 is assigned to N_2_; this is confirmed by the blank test (Figure S7b), which shows the same peak in the absence of any CO_2_. Gas chromatography–mass spectrometry (GC–MS) was also utilized for the detection of the evolved O_2_ from the photocatalytic CO_2_ reduction over F14‐SnS_2_. The peak located at *m*/*z* = 32 corresponds to O_2_, confirming the oxidative product formed during CO_2_ photoreduction into CO this confirms that carbon‐containing products do not originate from carbon contamination [40, 41].

Fourier‐transform infrared spectroscopy (FTIR) has been employed to identify reaction intermediates in the CO_2_ reduction process. In contrast to pristine SnS_2_, the F‐doped thin film exhibits distinct peaks under various reaction conditions (Figure 3b). When CO_2_ is adsorbed and activated on the surface, an absorption band corresponding to the monodentate carbonate (m‐CO_3_^2−^) around 1314 cm^−1^ are clearly visible after introducing CO_2_ and H_2_O vapor into the reaction without light irradiation [42]. Further, this peak becomes more intense when the lights are switched on, confirming that a subsequent reaction is occurring. Similarly, the carboxyl species (^*^COOH) peak around 1300 cm^−1^ strengthens more with elongated light illumination duration [43]. It was suggested that ^*^COOH is essential and acts as the rate‐determining intermediate in reducing CO_2_ to CO [10, 44, 45, 46]. In situ near ambient pressure X‐ray photoelectron spectroscopy (NAP‐XPS) has been utilized to analyze the C 1*s*, O 1*s*, Sn 3*d*, S 2*p*, and F 1*s* spectra of pristine and F‐doped SnS_2_ under various reaction conditions (labeled A to E). This analysis aimed to identify intermediates, clarify possible reaction pathways, and explore charge transfer (see Figure 3c; Figures S8–S10, along with Tables S5–S6) [47, 48]. The C 1*s* and O 1*s* spectra were fitted with six peaks (i.e., C─C, C─O, C═O, O─C═O, CO_3_^2−^, and CO_2_(g)) and four peaks (i.e., Si─O, CO_x_, H_2_O(g), and CO_2_(g)), respectively [47, 48]. The relative C─O/C─C, C═O/C─C, and O─C═O/C─C peak ratios and C─C, Sn 3*d*_5/2_, S 2*p*_3/2_, and F 1*s* peak positions are shown in Figure 3d,e, respectively. Notably, we did not include the CO_3_^2−^/C─C ratios, as they fell within the error margins of the fitting. Figure 3d illustrates that the C─O/C─C, C═O/C─C, and O─C═O/C─C peak ratios increase following the implantation of F into the SnS_2_ under different reaction conditions. This suggests that the stabilization of these intermediates is crucial for enhancing photocatalytic performance, ultimately leading to increased CO production. Figure 3e shows that the relative peak positions of Sn 3*d*_5/2_ and S 2*p*_3/2_ change similarly to that of the C─C peak for pristine SnS_2_. However, for the F14‐SnS_2_ a relative shift of 0.40, 0.41, and 0.59 eV was detected for the S 2*p*_3/2_, Sn 3*d*_5/2_ *_,_* and F 1*s* peaks, respectively. These shifts occurred in the presence of a CO_2_ + H_2_O atmosphere under light compared to ultra‐high vacuum (UHV) conditions. This indicates that F plays a significant role in facilitating charge transfer to the adsorbed CO_2_ and the reaction intermediates or activating neighboring elements.

### 2.4 Reaction Pathway

**FIGURE 4:**
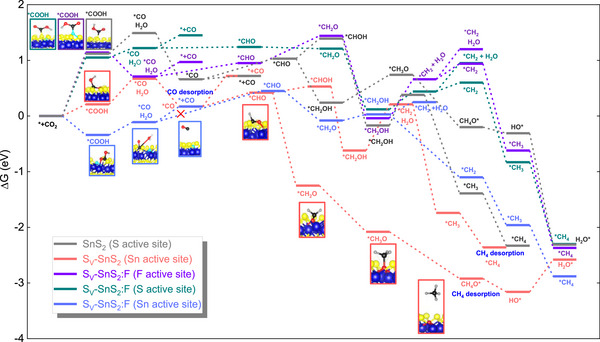
Reaction pathways on SnS_2_, S_V_‐SnS_2,_ and S_V_‐SnS_2_:F samples. Dark blue‐, yellow‐, cyan‐, black‐, red‐, and gray‐filled circles stand for Sn, S, F, C, O, and H atoms, respectively. SnS_2_ and S_V_‐SnS_2_ reaction pathways. Reproduced with permission [13] Copyright 2024, Elsevier.

To understand the photocatalytic production of CO from CO_2_ reaction mechanism on the surface of the S_V_‐SnS_2_:F photocatalyst, particularly on the SnS_2_(001) plane, has been systematically investigated by using the density functional theory (DFT) with Gibbs free energy (Δ*G*). This calculation serves as a descriptor that connects theoretical predictions with experimental measurements of catalytic activity. Our analysis identifies potential reaction pathways for five different systems: SnS_2_ with S as an active site, S_V_‐SnS_2_ with Sn as an active site, S_V_‐SnS_2_:F with F as an active site, S_V_‐SnS_2_:F with S as an active site, and S_V_‐SnS_2_:F with Sn as an active site (see Figure 4). The process begins with the adsorption of the CO_2_ molecule onto the surface, where it reacts with protons and electrons to form the first intermediate, ^*^COOH. The free‐energy profiles indicate that pristine SnS_2_ is limited by ^*^COOH formation, reflecting the poor CO_2_ activation capability of the basal surface. Introducing sulfur vacancies generates coordinatively unsaturated Sn siteseffectively modulating the local electronic structure, maintaining favorable ^*^COOH formation by lowering the energy barrier compared to SnS_2_, indicating that the CO_2_‐to‐^*^COOH pathway is exothermic and occurs spontaneously on S_V_‐SnS_2_.

On the other hand, the energy barrier for ^*^COOH formation on S_V_‐SnS_2_:F, where F and S serve as active sites, is higher; however, this energy barrier significantly decreases when Sn is the active site. Afterward, the ^*^COOH intermediate reacts with protons and electrons, H_2_O and ^*^CO are generated on the surface. This leads to the possibility of forming either CO(g) or the ^*^CHO intermediate following the reaction of CO with protons and electrons. Since Δ*G*_CO(g)_ > Δ*G*_CHO_, the formation of the CHO intermediate is favored in all systems, except for the S_V_‐SnS_2_:F sample, when S_V_ is connected to the F atom and the adjacent Sn serves as the active site.

Furthermore, DFT calculations indicate that the higher CO desorption energy (0.55 eV) compared with ^*^CHO formation (0.25 eV) suppresses CO poisoning and enables further hydrogenation of ^*^CO to ^*^CHO and subsequent intermediates, ultimately favoring CH_4_ formation in these systems.

In the S_V_‐SnS_2_:F sample wherein Sn, as the nearest neighbor of S_V_ connected to the F atom, serves as the active site, the CO desorption energy on S_V_–SnS_2_:F at the Sn active site is 0.28 eV (Table S7), which is substantially lower than the energy required for further hydrogenation of ^*^CO to ^*^CHO (0.45 eV). This energetic preference confirms that ^*^CO is more likely to desorb as CO(g) rather than undergo subsequent hydrogenation, thereby validating the facile CO release on the S_V_–SnS_2_:F surface. For comparison, on S_V_–SnS_2_, the higher CO desorption energy (0.55 eV) relative to ^*^CHO formation (0.25 eV) favors continued hydrogenation toward CH_4_. These results clearly demonstrate that F incorporation weakens ^*^CO binding and promotes CO desorption (Figure 4).

It should also be noted that, as shown in Table S1 and Figure S1, F doping and S‐vacancy formation induce modest changes in the bulk lattice parameters of SnS_2_, with variations within ∼0.2–0.3 Å. The free‐energy diagrams in Figure 4 reflect the impact of lattice changes associated with sulfur vacancies and F incorporation. Despite the small global lattice variations, clear differences in CO_2_ adsorption behavior and reaction energetics are observed among SnS_2_, S_V_‐SnS_2_, and S_V_‐SnS_2_:F. These differences mainly originate from local lattice distortion and electronic structure modulation at the active sites, which stabilize key intermediates and reduce the energy barriers for CO_2_ reduction, rather than from macroscopic lattice expansion or contraction.

Moreover, the probability (*P*) of finding the S_V_ close to an F atom can be estimated by using the relative formation energies and the Boltzmann distribution. That is, $P_{i}=Z^{-1}\;\times e^{-\frac{E_{f,i}}{k_{B}T}}$, where *E*_*f*,*i*_, *i k_B_*, *T*, and *Z* are the formation energy, Boltzmann constant, dummy index, temperature, and partition function, respectively (see Figures S11–S13, along with Tables S8–S10). Our calculations confirm the formation probability of S_V_–F pair is about 99% due to its low formation energy. Based on the calculated overall reaction pathway, along with in situ NAP‐XPS and in situ FTIR, F dopants play a crucial role in stabilizing the key and rate‐determining reaction intermediate ^*^COOH and enhancing the formation of CO thereon the nearby S_V_ and Sn sites.

## 3 Conclusion

In summary, we reported the effect of F ion doping into the SnS_2_ thin film prepared via the ion‐implantation method. Our result confirms that F atoms substitute at S sites, and the implantation process induces S_V_ due to the high kinetic energy impact of F ions on the SnS_2_ lattice. Both experimental observations and DFT calculations revealed a slight shrinkage in the crystal structure after the introduction of F into the SnS_2_ crystal structure. PC CO_2_ reduction experiments demonstrated that the optimal F incorporation of ∼7.2% F with ∼7.5% S_V_ switches the main product from CH_4_ (0.04 µmol cm^−2^) to CO (1.57 µmol cm^−2^). The F‐implanted sample also exhibited a high *IQE* of ∼0.52% and transfer electron rate *R_e_* of 2.2 e^−^ s^−1^ per active site (i.e., nearest‐neighbor Sn to F), which is among the best CO_2_ photocatalysts. Our experimental and theoretical results further unveiled that F dopant and S_V_ pairing are responsible for such a high PC performance by lowering the energy barrier of CO_2_ activation and stabilizing the ^*^COOH intermediate and product tuning.

## 4 Experimental Section

### 4.1 F‐Implanted SnS_2_ Preparation

F‐implanted SnS_2_ continuous thin film was grown by a three‐step sequential process, i.e., thermal evaporation of SnS_2_ powder (99.99%; Angene) precursor on the SiO_2_(300 nm)/Si substrate, ion‐implantation by an ion‐implanter equipped with multiple ion sources, and post‐sulfurization using sulphur powder (99.9%) as detailed in our previous work, and simulation of F ion implantation process was carried as detailed in Note S1. The F‐doped samples produced with different doses are designated as F14‐SnS_2_, F14‐SnS_2,_ and F15‐SnS_2_.

### 4.2 Measurements

The surface topology and thickness of films were assessed using an AFM (Bruker AXS) in non‐contact mode with an arrow‐type silicon AFM probe (Nano World; NCHR‐50) with a diameter of less than 16 nm. The measurement was controlled by a feedback mechanism. A Bruker D2 PHASER diffractometer with Cu K_α_ radiation (λ = 0.15406 nm) was used to measure thin film XRD. Diffraction angles were scanned from 2θ = 10° to 70° at a rate of 10° min^−1^. Raman spectra were taken using confocal HORIBA (iHR550) systems with green (532 nm) lasers. PL and TRPL. XPS measurements were performed using an Omicron XPS system with Al K_α_ X‐rays as the excitation source, at a voltage of 15 kV and 300 W. Binding energies were calibrated using C 1*s* at 284.6 eV. EPR studies were performed with a Bruker EMX‐Plus EPR spectrometer. The Taiwan National Synchrotron Radiation Research Center (NSRRC) facility beam lines TPS 44A1 and TLS 16A1 were used to measure XAS spectra for the XANES of Sn and S K‐edges. The XAS data were processed with Athena software [49]. HRTEM, operated at 100 kV (JEM–ARM200FTH), was used to record the microstructures of SnS_2_ and S_V_‐SnS_2_:F. The HRTEM images were analyzed using the Gatan Microscopy Suite (GMS3) software. The absorption spectrum was measured with a Jasco V‐670 double‐beam spectrophotometer. The TAS data was acquired using a commercial transient absorption spectrometer (Femto Frame II, IB Photonics). An 800 nm, 1 kHz, 100 fs amplified pulsed laser was generated by the Spitfire (Spectra Physics) using a mode‐locked Ti: Sapphire laser (Tsunami, Spectra Physics) as the seed laser. An optical parametric amplifier (OPA, TOPAS‐C, Light Conversion) provided tunable pump wavelengths; for this study, a pump wavelength of 400 nm was selected. The pump and probe beams were focused on the same position on the sample, with beam diameters of 300 and 100 µm, respectively, to capture the transient absorption spectra. A broadband white‐light probe (450–700 nm) with a pulse duration of 30 fs, generated by supercontinuum generation in a thick sapphire plate, was utilized for the measurement. The decay kinetics of photogenerated electrons were studied using global fitting analysis spanning the wavelength range 600–700 nm (at 10 nm intervals).

### 4.3 Photocatalytic CO_2_ Reduction

PC CO_2_ reduction experiments were conducted in a custom‐built 20.0 mL stainless steel reactor. Prior to the experiment, the reactor was degassed at 100 °C and purged with nitrogen to remove residual compounds, gases, and impurities. CO_2_ gas was then introduced into the reactor via water at a flow rate of 45 sccm for 15 min, followed by 5 sccm for another 15 min to maintain consistent humidity and ensure equilibrium gas adsorption/desorption. The light source was a 150 W commercial halogen lamp with AM1.5, which was positioned directly above the catalysts. The reaction was carried out for 4 h under 1 sun irradiance. The CH_4_ and CH_3_CHO signals were recorded by injecting 0.5 mL of the products (using a Trajan SGE syringe) into a gas chromatography system (GC; Agilent 6890 GC) equipped with a flame ionization detector (FID) set at 150 °C and a glass column (Porapak Q, 80–100 mesh). The injection and oven temperatures were maintained at 50 °C and 105 °C, respectively. Notably, the selectivity (%) of a product was computed using *S* (%) = 100 × *Y_i_*/*Y_t_* , where *Y_i_* represents the yield of product *i* (with *i* being either methane, carbon monoxide, or acetaldehyde), and *Y_t_* denotes the total products yield. Additionally, the ^13^CO_2_ isotope test was done using an RT‐Msieve 5A column (15 m, I.D.: 0.25 mm) at 35 °C, with helium as the carrier gas flowing at a rate of 1 mL min^−1^. All measurements were repeated at least three times independently, and the experimental uncertainty is reported as the standard deviation of these measurements and shown as error bars in the corresponding figures.

### 4.4 In Situ FTIR Measurements

The in situ FTIR measurement was carried out with a custom‐built polarization modulation infrared reflection absorption spectroscopy accessory. This accessory was integrated with an FTIR spectrometer equipped with a photoelastic modulator (PEM‐200, Hinds Instruments) and a HgCdTe (MCT) detector. Final spectra were obtained by subtracting the reference spectrum from all recorded infrared spectra. Each spectrum was recorded over a 10‐min period.

### 4.5 In Situ Near Ambient Pressure XPS Measurements

The NSRRC facility beam line TLS 24A1 was utilized to carry out NAP‐XPS experiments. Initially, XPS spectra of Sn *3d*, S *2p*, F *1s*, C *1s*, O *1s*, and Au *4f* were obtained under UHV conditions. The sample was then heated to 400 K for 30 min before being allowed to cool naturally to room temperature. XPS spectra were then collected at the same site, known as condition (A) UHV. In situ NAP‐XPS spectra were also collected under four different conditions: (B) in a CO_2_ atmosphere in the dark, (C) in a 1 mbar CO_2_ atmosphere under light illumination, (D) in a 1 mbar CO_2_ + 1 mbar H_2_O atmosphere in the dark, and (E) in a 1 mbar CO_2_ + 1 mbar H_2_O atmosphere under light illumination. The NAP‐XPS spectra were processed with Casa XPS software (version 2.3.20), which applied the Shirley background correction to all recorded spectra. The binding energies were calibrated using Au 4*f*_7/2_ peaks at 84.0. NAP‐XPS data analyses and fitting processes can be found in our previous report [13].

### 4.6 Computational Calculations

In this study, we performed the possible ways of introducing a fluorine (F) doping atom into the 2D SnS_2_ trigonal $P\bar{3}m1$ structure with formation energy, electronic properties, and CO_2_ reduction mechanism by the DFT calculations using the Vienna ab initio simulation package (VASP) [50]. The generalized gradient approximation (GGA) in the formalism of Perdew–Burke–Ernzerhof (PBE) was adopted to describe the electronic exchange‐correlation energy. The projector augmented wave (PAW) pseudo‐potentials were chosen to describe ionic cores [51, 52]. We have utilized the standard PBE functional method to improve the van der Waals (vdW) interactions correctly by Becke‐Jonson damping potential with the DFT‐D3 method [53]. The valence electron configurations considered in this calculation are Sn, F, S, C, and O. The convergence criterion for the self‐consistent iteration was 10^−5^ eV. The calculations were carried out with a plane‐wave basis set, and the cutoff energy was set to 520 eV with a Gaussian smearing method of 0.05 eV width, in order to assure well‐converged total energy and force values. In here, the structural optimization of layered 2‐D trigonal SnS_2_, belonging to a $P\bar{3}m1$ space group, was carried out using a 7 × 7 × 3 Γ‐centered mesh of *k*‐points. The calculated lattice parameters, *a* = 3.683 Å and *c* = 6.048 Å, closely matches the experimental values (*a* = 3.649 Å and *c* = 5.899 Å) [54, 55]. To model the F‐doped material, we used a 4 × 4 supercell of SnS_2_, which contains 32 units and has a cell volume of 14.727 × 14.727 × 11.939 Å^3^. We explored two methods for introducing the F atom into the SnS_2_ lattice. The first method involves substituting F for either S or Sn atoms (denoted as F@S and F@Sn, respectively), while the second involves placing the F atom in an interstitial position between the layers (denoted as F@int). We calculated the absolute formation energies (*E_f_*) of various configurations using a specific relation, with the aim of assessing the stability of the dopant.

(1) $$E_{f}=E\left(SnS_{2}:F\right)-E\left(SnS_{2}\right)-\mu \left(F\right)+\mu \left(S\right)$$

(2) $$E_{f}=E\left(SnS_{2}:F\right)-E\left(SnS_{2}\right)-\mu \left(F\right)+\mu \left(Sn\right)$$

(3) $$E_{f}=E\left(SnS_{2}:F\right)-E\left(SnS_{2}\right)-\mu \left(F\right)$$

For the substitution of F in the S site (Equation 1), the substitution of F in the Sn site (Equation 2), and the interstitial doping of F between the layers (Equation 3), respectively. We denote the total energies of the pristine SnS_2_ supercell and the F‐doped SnS_2_ as, *E*(*SnS*_2_) and *E*(*SnS*_2_: *F*), respectively. The quantities μ(*F*), μ(*S*), and μ(*Sn*), represent the chemical potentials of F, substituted S, and substituted Sn atoms in the host lattice, respectively. The chemical potentials for doping are calculated from black fluorine, α‐Solid S_8_, and bulk Sn metal, respectively. In here, the lattice optimization was performed with full relaxation until the residual forces were below 0.05 eV Å^−2^. The results show that F substitution at the S site causes minimal lattice distortion (< 0.1%), whereas substitution at the Sn site, interstitial F, and vacancy defects lead to more pronounced changes, especially along the *c*‐axis (up to ∼1% expansion or ∼1.6% contraction for Sn vacancy). This indicates that F substitution at the S site is structurally the most compatible and is consistent with its lower formation energy. Further calculations using the screened hybrid Heyd–Scuseria–Ernzerh of (HSE) functional (mixing parameter was 0.3) without extra relaxation were carried out to obtain a total and projected density of states (DOS and PDOS) [56, 57]. Atomic charges of the optimized structures were calculated using the Bader method with a program designed by Henkelman et al. [58]. We also calculate the isosurfaces of charge density difference analysis, which shows that apparent electron transfers occur from the F to SnS_2_. For free energy diagrams for CO_2_ reduction mechanism, the Gibbs free energies for each gaseous and adsorbed species were calculated at 298.15 K. VESTA (3.5.7) software was used to draw the relaxed configurations [59].

## Author Contributions

T.T.M. conceived the idea of the study, designed the experiments, prepared the samples, recorded in situ NAP‐XPS spectra, analyzed XPS, in situ NAP‐XPS, in situ FTIR and Raman, conducted UV–visible, GC experiments, analyzed the results, and wrote the first draft and revised the manuscript. M.Q. contributed to the conception of the study and designed the experiments, analyzing XPS and in situ NAP‐XPS spectra, and revising the manuscript. A.S. performed HRTEM and EDX. T.T.M. and A.G.H. carried out XAS and XRD. P.R. and M.‐C.L. performed the DFT simulations. Y.‐Y.W. and Y.‐J.L. carried out and analyzed TAS. C.‐M.C. and W.‐Y.W. contributed to carrying out ion implantation. S‐Y.C. recorded TRPL.W.‐H.Y. and H.‐L.W. carried out and analyzed in situ FTIR. K.‐T.W., K.‐H.C., and L.‐C.C. contributed to project administration and funding acquisition, supervised the research, conceived the idea of the study, and revised the manuscript. All authors discussed the results and commented on the manuscript.

## Funding

This work was supported by the National Science and Technology Council (NSTC) of Taiwan, Academic Summit Project (NSTC 112‐2639‐M‐002‐005‐ASP, NSTC 113‐2639‐M‐002‐004‐ASP, NSTC 114‐2639‐M‐002‐003‐ASP, NSTC 112‐2113‐M‐001‐023, NSTC 113‐2113‐M‐001‐012, and NSTC 114‐2112‐M‐003‐019‐MY2). Additional financial support from the Center of Atomic Initiative for New Materials, National Taiwan University, from the Featured Areas Research Center Program within the Framework of the Higher Education Sprout Project by the Ministry of Education in Taiwan (112L9008, 113L9008, and 114L9008), and Academia Sinica (AS‐SS‐112‐01 and AS‐TP‐113‐M02) are also acknowledged.

## Conflicts of Interest

The authors declare no conflicts of interest.

## Supporting information




**Supporting File**: advs73956‐sup‐0001‐SuppMat.docx.

## Data Availability

The data that support the findings of this study are available from the corresponding author upon reasonable request.

## References

[1] J. Michl , “Towards an Artificial Leaf?,” Nature Chemistry 3 (2011): 268–269, 10.1038/nchem.1021. 21430681

[2] M. Qorbani , A. Sabbah , Y. R. Lai , et al., “Atomistic Insights Into Highly Active Reconstructed Edges of Monolayer 2H‐WSe_2_ Photocatalyst,” Nature Communications 13 (2022): 1–8, 10.1038/s41467-022-28926-0. PMC8913837 35273184

[3] Ž. Kovačič , B. Likozar , and M. Huš , “Photocatalytic CO_2_ Reduction: A Review of Ab Initio Mechanism, Kinetics, and Multiscale Modeling Simulations,” ACS Catalysis 10 (2020): 14984–15007, 10.1021/acscatal.0c02557.

[4] I. Roger , M. A. Shipman , and M. D. Symes , “Earth‐abundant Catalysts for Electrochemical and Photoelectrochemical Water Splitting,” Nature Reviews Chemistry 1 (2017): 0003, 10.1038/s41570-016-0003.

[5] C. Rosso , G. Filippini , A. Criado , M. Melchionna , P. Fornasiero , and M. Prato , “Metal‐Free Photocatalysis: Two‐Dimensional Nanomaterial Connection Toward Advanced Organic Synthesis,” ACS Nano 15 (2021): 3621–3630, 10.1021/acsnano.1c00627. 33715354 PMC8041367

[6] F. Dong , J. Sheng , Y. He , et al., “Identification of Halogen‐associated Active Sites on Bismuth‐Based Perovskite Quantum Dots for Efficient and Selective CO_2_‐to‐CO Photoreduction,” ACS Nano 14 (2020): 13103–13114, 10.1021/acsnano.0c04659. 32940453

[7] F. Y. Fu , I. Shown , C. S. Li , et al., “KSCN‐Induced Interfacial Dipole in Black TiO_2_ for Enhanced Photocatalytic CO_2_ Reduction,” ACS Applied Materials & Interfaces 11 (2019): 25186–25194, 10.1021/acsami.9b06264. 31268648

[8] P.‐P. Huang , M. Qorbani , Y.‐T. Hung , et al., “Capped Vapor–Liquid–Solid Growth of Vanadium‐Substituted Molybdenum Disulfide Ultrathin Films for Enhanced Photocatalytic Activity,” ACS Nano (2026), 10.1021/acsnano.5c17367. PMC12825380 41498384

[9] M. K. Hussien , A. Sabbah , M. Qorbani , et al., “Constructing B─N─P Bonds in Ultrathin Holey g‐C_3_N_4_ for Regulating the Local Chemical Environment in Photocatalytic CO_2_ Reduction to CO,” Small 20 (2024): 1–14, 10.1002/smll.202400724. 38639018

[10] N. Q. Thang , A. Sabbah , R. Putikam , et al., “Regulating COOH Intermediate via Rationally Constructed Surface‐Active Sites of Bi_2_WO_6_ for Solar‐Driven CO_2_ ‐to‐CO Production,” Advanced Functional Materials 35 (2025): 1–13, 10.1002/adfm.202423751.

[11] Y. J. Jang , I. Jeong , J. Lee , J. Lee , M. J. Ko , and J. S. Lee , “Unbiased Sunlight‐Driven Artificial Photosynthesis of Carbon Monoxide From CO_2_ Using a ZnTe‐Based Photocathode and a Perovskite Solar Cell in Tandem,” ACS Nano 10 (2016): 6980–6987, 10.1021/acsnano.6b02965. 27359299

[12] I. Shown , S. Samireddi , Y. C. Chang , et al., “Carbon‐doped SnS_2_ Nanostructure as a High‐efficiency Solar Fuel Catalyst Under Visible Light,” Nature Communications 9 (2018): 169, 10.1038/s41467-017-02547-4. PMC5766557 29330430

[13] T. T. Mamo , M. Qorbani , A. G. Hailemariam , et al., “Enhanced CO_2_ Photoreduction to CH_4_ via *COOH and *CHO Intermediates Stabilization by Synergistic Effect of Implanted P and S Vacancy in Thin‐Film SnS_2_ ,” Nano Energy 128 (2024): 109863, 10.1016/j.nanoen.2024.109863.

[14] T. Billo , I. Shown , T. Amerta , et al., “A Mechanistic Study of Molecular CO2 Interaction and Adsorption on Carbon Implanted SnS_2_ Thin Film for Photocatalytic CO_2_ Reduction Activity,” Nano Energy 72 (2020): 104717, 10.1016/j.nanoen.2020.104717.

[15] S. Yin , X. Zhao , E. Jiang , Y. Yan , P. Zhou , and P. Huo , “Boosting Water Decomposition by Sulfur Vacancies for Efficient CO_2_ Photoreduction,” Energy & Environmental Science 15 (2022): 1556–1562, 10.1039/d1ee03764a.

[16] L. Dong , J. Yang , X. Xu , et al., “Effect of Fluorine Ion Irradiation on the Properties of Monolayer Molybdenum Disulfide,” Journal of Applied Physics 132 (2022): 225107, 10.1063/5.0114012.

[17] W. Yu , X. Liu , L. Pan , et al., “Enhanced Visible Light Photocatalytic Degradation of Methylene Blue by F‐doped TiO_2_ ,” Applied Surface Science 319 (2014): 107–112, 10.1016/j.apsusc.2014.07.038.

[18] G. Vitiello , G. Iervolino , C. Imparato , et al., “F‐Doped ZnO Nano‐ and Meso‐Crystals With Enhanced Photocatalytic Activity in Diclofenac Degradation,” Science of The Total Environment 762 (2021): 143066, 10.1016/j.scitotenv.2020.143066. 33127133

[19] J. Liang , L. Liang , B. Zeng , et al., “Fluorine‐Doped Carbon Support Enables Superfast Oxygen Reduction Kinetics by Breaking the Scaling Relationship,” Angewandte Chemie International Edition 63 (2024), 202412825, 10.1002/anie.202412825. 39119836

[20] X. Yan , C. Chen , Y. Wu , et al., “Boosting CO_2_ Electroreduction to C_2_ ^+^ Products on Fluorine‐Doped Copper,” Green Chemistry 24 (2022): 1989–1994, 10.1039/d1gc04824d.

[21] J. Xie , X. Zhao , M. Wu , Q. Li , Y. Wang , and J. Yao , “Metal‐Free Fluorine‐Doped Carbon Electrocatalyst for CO_2_ Reduction Outcompeting Hydrogen Evolution,” Angewandte Chemie International Edition 57 (2018) 9640–9644, 10.1002/anie.201802055. 29611887

[22] D. Yuan , Z. Wei , P. Han , et al., “Electron Distribution Tuning of Fluorine‐Doped Carbon for Ammonia Electrosynthesis,” Journal of Materials Chemistry A 7 (2019): 16979–16983, 10.1039/c9ta04141a.

[23] S. G. Han , D. D. Ma , S. H. Zhou , et al., “Fluorine‐tuned Single‐atom Catalysts With Dense Surface Ni‐N_4_ Sites on Ultrathin Carbon Nanosheets for Efficient CO_2_ Electroreduction,” Applied Catalysis B: Environmental 283 (2021): 119591, 10.1016/j.apcatb.2020.119591.

[24] H. S. Rupprecht and A. E. Michel , “Trends of Ion Implantation in Silicon Technology,” Ion Implantation and Beam Processing (1984): 311–326, 10.1016/b978-0-12-756980-2.50013-8.

[25] Y. Kayser , P. Hönicke , D. Banas̈ , et al., “Depth Profiling of Low Energy Ion Implantations in Si and Ge by Means of Micro‐Focused Grazing Emission X‐Ray Fluorescence and Grazing Incidence X‐Ray Fluorescence,” Journal of Analytical Atomic Spectrometry 30 (2015): 1086–1099, 10.1039/c4ja00461b.

[26] U. Bangert , W. Pierce , D. M. Kepaptsoglou , et al., “Ion Implantation of Graphene—Toward IC Compatible Technologies,” Nano Letters 13 (2013): 4902–4907, 10.1021/nl402812y. 24059439

[27] H. Zhang , L. Mao , J. Wang , et al., “One‐Step Fabricated Sn^0^ Particle on S‐Vacancies SnS_2_ to Accelerate Photoelectron Transfer for Sterling Photocatalytic CO_2_ Reduction in Pure Water Vapor Environment,” Small 20 (2023): 1–10, 10.1002/smll.202305727. 37699770

[28] A. G. Hailemariam , M. Qorbani , T. T. Mamo , et al., “Improved Electrochemical Kinetics and Rate Performance of Lithium‐Ion Batteries by Li_2_ FeS_2‐x_ F_x_ Cathode Materials,” Communications Materials 6 (2025): 1–11.

[29] T. Qiang , L. Chen , Y. Xia , and X. Qin , “Dual Modified MoS_2_/SnS_2_ Photocatalyst With Z‐Scheme Heterojunction and Vacancies Defects to Achieve a Superior Performance in Cr (VI) Reduction and Dyes Degradation,” Journal of Cleaner Production 291 (2021): 125213, 10.1016/j.jclepro.2020.125213.

[30] W. Wu and L. Yang , “Spin Polarization Engineering on SnS_2_ for Improving Photocatalytic Disinfection,” Journal of Catalysis 429 (2024): 115283, 10.1016/j.jcat.2023.115283.

[31] A. M. Panich , A. I. Shames , R. E. Abutbul , N. Maman , S. D. Goren , and Y. Golan , “NMR and EPR Study of Cubic π‐Phase SnS Semiconductor Nanoparticles,” Materials Chemistry and Physics 250 (2020): 123206, 10.1016/j.matchemphys.2020.123206.

[32] V. Popovych , M. Bester , I. Stefaniuk , and M. Kuzma , “Dyson Line and Modified Dyson Line in the EPR Measurements,” Nukleonika 60 (2015): 385–388, 10.1515/nuka-2015-0068.

[33] M. Ma , G. Liu , Z. Yang , and G. Zhang , “Biaxial Strain Modulation of the Optoelectronic Properties of F‐Doped Defective Monolayer SnS_2_ ,” Physica Scripta 99 (2024): 055935, 10.1088/1402-4896/ad36f9.

[34] J. Klein , L. Kampermann , B. Mockenhaupt , M. Behrens , J. Strunk , and G. Bacher , “Limitations of the Tauc Plot Method,” Advanced Functional Materials 33 (2023): 1–19, 10.1002/adfm.202304523.

[35] H. Wang , D. Yong , S. Chen , et al., “Oxygen‐Vacancy‐Mediated Exciton Dissociation in Biobr for Boosting Charge‐Carrier‐Involved Molecular Oxygen Activation,” Journal of the American Chemical Society 140 (2018): 1760–1766, 10.1021/jacs.7b10997. 29319310

[36] J. Sun , Y. Yang , J. I. Khan , et al., “Ultrafast Carrier Trapping of a Metal‐doped Titanium Dioxide Semiconductor Revealed by Femtosecond Transient Absorption Spectroscopy,” ACS Applied Materials & Interfaces 6 (2014): 10022–10027, 10.1021/am5026159. 24918499

[37] P. Zhang , T. Tachikawa , M. Fujitsuka , and T. Majima , “In Situ Fluorine Doping of TiO_2_ Superstructures for Efficient Visible‐Light Driven Hydrogen Generation,” Chemsuschem 9 (2016): 617–623, 10.1002/cssc.201501558. 26871554

[38] H. Sun , K. Wei , D. Wu , et al., “Structure Defects Promoted Exciton Dissociation and Carrier Separation for Enhancing Photocatalytic Hydrogen Evolution,” Applied Catalysis B: Environmental 264 (2020): 118480, 10.1016/j.apcatb.2019.118480.

[39] W. Yang , L. Zhang , J. Xie , et al., “Enhanced Photoexcited Carrier Separation in Oxygen‐Doped ZnIn_2_S_4_ Nanosheets for Hydrogen Evolution,” Angewandte Chemie International Edition 55 (2016): 6716–6720, 10.1002/anie.201602543. 27100950

[40] M. Kamal Hussien , A. Sabbah , M. Qorbani , et al., “Metal‐free Four‐in‐one Modification of G‐C_3_N_4_ for Superior Photocatalytic CO_2_ Reduction and H_2_ Evolution,” Chemical Engineering Journal 430 (2022): 132853, 10.1016/j.cej.2021.132853.

[41] A. Sabbah , I. Shown , M. Qorbani , et al., “Boosting Photocatalytic CO_2_ Reduction in a ZnS/ZnIn_2_S_4_ Heterostructure Through Strain‐Induced Direct Z‐Scheme and a Mechanistic Study of Molecular CO_2_ Interaction Thereon,” Nano Energy 93 (2022): 106809, 10.1016/j.nanoen.2021.106809.

[42] Z. Jiang , H. Sun , T. Wang , et al., “Nature‐Based Catalyst for Visible‐Light‐Driven Photocatalytic CO_2_ Reduction,” Energy & Environmental Science 11 (2018): 2382–2389, 10.1039/c8ee01781f.

[43] N. J. Firet and W. A. Smith , “Probing the Reaction Mechanism of CO_2_ Electroreduction Over Ag Films via Operando Infrared Spectroscopy,” ACS Catalysis 7 (2017): 606–612, 10.1021/acscatal.6b02382.

[44] X. Zhu , H. Xu , J. Liu , et al., “Stacking Engineering of Heterojunctions in Half‐Metallic Carbon Nitride for Efficient CO_2_ Photoreduction,” Advanced Science 10 (2023): 1–12, 10.1002/advs.202307192. PMC10754085 38072660

[45] J. Li , Q. Chai , R. Niu , et al., “Identification of Intrinsic Vacancies and Polarization Effect on Ternary Halo‐Sulfur‐Bismuth Compounds for Efficient CO_2_ Photoreduction Under Near‐Infrared Light Irradiation,” Carbon Energy 6 (2024): 598, 10.1002/cey2.598.

[46] Q. Lin , J. Zhao , P. Zhang , et al., “Highly Selective Photocatalytic Reduction of CO_2_ to CH_4_ on Electron‐Rich Fe Species Cocatalyst Under Visible Light Irradiation,” Carbon Energy 6 (2024): 435, 10.1002/cey2.435.

[47] L. Collado , A. Reynal , F. Fresno , et al., “Unravelling the Effect of Charge Dynamics at the Plasmonic Metal/Semiconductor Interface for CO_2_ Photoreduction,” Nature Communications 9 (2018): 1–10, 10.1038/s41467-018-07397-2. PMC6255847 30478316

[48] M. Favaro , H. Xiao , T. Cheng , W. A. Goddard , and E. J. Crumlin , “Subsurface Oxide Plays a Critical Role in CO_2_ Activation by Cu(111) Surfaces to Form Chemisorbed CO_2_, the First Step in Reduction of CO_2_ ,” Proceedings of the National Academy of Sciences 114 (2017): 6706–6711, 10.1073/pnas.1701405114. PMC5495248 28607092

[49] B. Ravel and M. Newville , “ATHENA , ARTEMIS , HEPHAESTUS: Data Analysis for X‐Ray Absorption Spectroscopy Using IFEFFIT,” Journal of Synchrotron Radiation 12 (2005): 537–541, 10.1107/S0909049505012719. 15968136

[50] G. Kresse and J. Furthmüller , “Efficient Iterative Schemes for Ab Initio Total‐Energy Calculations Using a Plane‐Wave Basis Set,” Physical Review B 54 (1996): 11169–11186, 10.1103/PhysRevB.54.11169. 9984901

[51] J. P. Perdew , K. Burke , and M. Ernzerhof , “Generalized Gradient Approximation Made Simple,” Physical Review Letters 77 (1996): 3865–3868, 10.1103/PhysRevLett.77.3865. 10062328

[52] C. Lee , W. Yang , and R. G. Parr , “Development of the Colle‐Salvetti Correlation‐Energy Formula Into a Functional of the Electron Density,” Physical Review B 37 (2011): 785–789, 10.1103/PhysRevB.37.785. 9944570

[53] A. Tkatchenko , R. A. Distasio , R. Car , and M. Scheffler , “Accurate and Efficient Method for Many‐Body van der Waals Interactions,” Physical Review Letters 108 (2012): 1–5, 10.1103/PhysRevLett.108.236402. 23003978

[54] L. A. Burton , T. J. Whittles , D. Hesp , et al., “Electronic and Optical Properties of Single Crystal SnS_2_: An Earth‐abundant Disulfide Photocatalyst,” Journal of Materials Chemistry A 4 (2016): 1312–1318, 10.1039/c5ta08214e.

[55] N. V. Podberezskaya , S. A. Magarill , N. V. Pervukhina , and S. V. Borisov , “Crystal Chemistry of Dichalcogenides MX_2_ ,” Journal of Structural Chemistry 42 (2001): 654–681, 10.1023/A:1013106329156.

[56] J. Heyd , G. E. Scuseria , and M. Ernzerhof , “Hybrid Functionals Based on a Screened Coulomb Potential,” The Journal of Chemical Physics 118 (2003): 8207–8215, 10.1063/1.1564060.

[57] A. V. Krukau , O. A. Vydrov , A. F. Izmaylov , and G. E. Scuseria , “Influence of the Exchange Screening Parameter on the Performance of Screened Hybrid Functionals,” The Journal of Chemical Physics 125 (2006): 1–5, 10.1063/1.2404663. 17176133

[58] G. Henkelman , A. Arnaldsson , and H. Jónsson , “A Fast and Robust Algorithm for Bader Decomposition of Charge Density,” Computational Materials Science 36 (2006): 354–360, 10.1016/j.commatsci.2005.04.010.

[59] K. Momma and F. Izumi , “VESTA 3 for Three‐Dimensional Visualization of Crystal, Volumetric and Morphology Data,” Journal of Applied Crystallography 44 (2011): 1272–1276, 10.1107/S0021889811038970.

