# Supplementary material for: Basal Plane Activation of SnS2 Thin‐Film by Fluorine Doping for Selective Solar‐Driven CO2 Reduction With Enhanced Quantum Efficiency
Source: Adv Sci (Weinh). 2026 Jan 20;13(21):e22924. doi: 10.1002/advs.202522924 (PMC13073233; doi:10.1002/advs.202522924)
Supplement: Supplementary file 1 — Supporting File: advs73956‐sup‐0001‐SuppMat.docx. [file ADVS-13-e22924-s001.docx]

Supporting Information

**Basal Plane Activation of SnS_2_ Thin-Film by Fluorine Doping for Selective Solar-Driven CO_2_ Reduction with Enhanced Quantum Efficiency**

*Tadios Tesfaye Mamo, Mohammad Qorbani*, Adane Gebresilassie Hailemariam, Putikam Raghunath, Che-Men Chu, Wen-Hsin Yuan, Amr Sabbah, Yen-Yu Wang, Shuo-Yun Chang, Ming-Chang Lin, Wei-Yen Woon, Yu-Jung Lu, Heng-Liang Wu*, Ken-Tsung Wong, Kuei-Hsien Chen*, and Li-Chyong Chen**

T.T. Mamo, A.G. Hailemariam, W.-H. Yuan, A. Sabbah, H.-L. Wu, K.-H. Chen, L.-C. Chen

Center for Condensed Matter Sciences, National Taiwan University, Taipei, 10617, Taiwan
E-mail: hengliangwu@ntu.edu.tw; [chenkh@pub.iams.sinica.edu.tw](mailto:chenkh@pub.iams.sinica.edu.tw); [chenlc@ntu.edu.tw](mailto:chenlc@ntu.edu.tw)

T.T. Mamo, A. Sabbah, H.-L. Wu, L.-C. Chen

Center of Atomic Initiative for New Materials, National Taiwan University, Taipei, 10617, Taiwan

T.T. Mamo, W.-H. Yuan, H.-L. Wu, K.-T. Wong

Department of Chemistry, National Taiwan University, Taipei, 10617, Taiwan

T.T. Mamo, S.-Y. Chang, K.-T. Wong, K.-H. Chen

Institute of Atomic and Molecular Sciences, Academia Sinica, Taipei, 10617, Taiwan

M. Qorbani

Undergraduate Program of Electro-Optical Engineering, National Taiwan Normal University, Taipei, 11677 Taiwan

E-mail: [qorbani@ntnu.edu.tw](mailto:qorbani@ntnu.edu.tw)

M. Qorbani

Institute of Electro-Optical Engineering, National Taiwan Normal University, Taipei 11677, Taiwan

P. Raghunath, M.-C. Lin

Department of Applied Chemistry, National Yang Ming Chiao Tung University, Hsinchu 30010, Taiwan

A.G. Hailemariam

Department of Chemistry, College of Natural Science, Dire Dawa University, Dire Dawa 1362, Ethiopia

C.-M. Chu, W.-Y. Woon

Department of Physics, National Central University, Jungli 32054, Taiwan

1. Sabbah

Tabbin Institute for Metallurgical Studies, Tabbin, Helwan 109, Cairo, 11421, Egypt

Y.-Y. Wang, Y.-J. Lu, L.-C. Chen

Department of Physics, National Taiwan University, Taipei, 10617, Taiwan

Y.-Y. Wang, Y.-J. Lu

Research Center for Applied Sciences, Academia Sinica, Taipei, 11529, Taiwan

S.-Y. Chang

Department of Chemistry, National Tsing-Hua University, Hsinchu, 30013, Taiwan

H.-L. Wu

National Synchrotron Radiation Research Center, Hsinchu 30092, Taiwan

Contents

[Supplementary Figures 4](#_Toc218543310)

[Figure S1. Configurations of F-doped SnS_2_ by DFT calculation. 4](#_Toc218543311)

[Figure S2. Bader charge analysis. 5](#_Toc218543312)

[Figure S3. Partial density of states with respective configurations. 5](#_Toc218543313)

[Figure S4. UPS data. 6](#_Toc218543314)

[Figure S5. Crystal structure after PC stability test. 7](#_Toc218543315)

[Figure S6. Lattice vibrational mode after photocatalytic stability test. 8](#_Toc218543316)

[Figure S7. Isotope experiments. 9](#_Toc218543317)

[Figure S8. Normalized NAP-XPS signal versus relative binding energy of the grown thin films. 10](#_Toc218543318)

[Figure S9. NAP-XPS spectra of the grown thin films. 11](#_Toc218543319)

[Figure S10. VBM of NAP-XPS of the grown thin films under different environments. 12](#_Toc218543320)

[Figure S11. Configurations of F-doped in different S sites of SnS_2_:F by DFT calculations. 13](#_Toc218543321)

[Figure S12. Configurations of S_V_ in different S sites of S_V_ -SnS2 by DFT calculations. 14](#_Toc218543322)

[Figure S13. Configurations of S_V_ in different S sites of SnS_2_:F by DFT calculations. 15](#_Toc218543323)

[Supplementary Tables 16](#_Toc218543324)

[Table S1. Formation energy of F dopant in SnS_2_. 16](#_Toc218543325)

[Table S2. XPS fitted peak positions for SnS_2_ and F14-SnS_2_ films.. 16](#_Toc218543326)

[Table S3. Calculation details of $AQE$ and $IQE$. 17](#_Toc218543327)

[Table S4. Photocatalytic CO_2_ reduction productivity comparison. 18](#_Toc218543328)

[Table S5. In-situ NAP-XPS fitted peak position for Sn 3*d*_5/2_, S 2*p*_3/2_, and F 1*s* elements of SnS_2_ and F-SnS_2_ samples. 20](#_Toc218543329)

[Table S6. In-situ NAP-XPS fitted peak position for C 1*s*, and O 1*s* elements of SnS_2_ and F-SnS_2_ samples. 21](#_Toc218543330)

[Table S7. Desorption energy of CO.. 22](#_Toc218543331)

[Table S8. Relative formation energy of F in different S sites of SnS_2_:F. 23](#_Toc218543332)

[Table S9. Relative formation energy of S_V_ in different S sites of S_V_-SnS_2_. 24](#_Toc218543333)

[Table S10. Relative formation energy of S_V_ in different S sites of SnS_2_:F. 24](#_Toc218543334)

[Supplementary Notes 25](#_Toc218543335)

[Note S1. Growth of F-implanted SnS_2_ thin film 25](#_Toc218543336)

[Figure N1. SRIM simulation. 25](#_Toc218543337)

[Supplementary References 26](#_Toc218543338)

#

# Supplementary Figures

Figure S1. Configurations of F-doped SnS_2_ by DFT calculation. a–h) Side-view of the calculated configurations of the SnS_2_ ($P\bar{3}m1$), SnS_2_ (F-sub. @S), SnS_2_ (F-sub. @Sn), SnS_2_ (F @ int.-1), SnS_2_ (F @ int.-2), SnS_2_ (F @ int.-3), SnS_2_ - S_V_, and SnS_2_ –Sn_V_, respectively. Dark blue-, yellow- and cyan-filled circles stand for Sn, S, and F atoms, respectively. Red-filled circles show S and Sn defects. Structures enclosed with border lines represent periodic calculation models.

Figure S2. Bader charge analysis. a,b) Bader charge of S_V_-SnS_2_, and S_V_-SnS_2_:F, respectively. Dark blue-, yellow-, and cyan-filled circles stand for Sn, S, and F atoms, respectively. Dark Orange circle shows S_V._

Figure S3. Partial density of states with respective configurations. a,b) Configurations of 2F-doped SnS_2_ and its PDOS, respectively. c,d) Configurations of 3F-doped SnS_2_ and its PDOS, respectively.

Figure S4. UPS data. a,b) UPS spectra taken with a photon energy of 21.2 eV of SnS_2_ and F14-SnS_2_, respectively Insets illustrate the zoomed-in views of the low binding energy region. c,d) LEIPS curves of SnS_2_ and F14-SnS_2_, respectively.

Figure S5. Crystal structure after PC stability test. XRD peak of the F14-SnS_2_ sample after stability test. Our observation indicates that the peak positions of the film remained unchanged after the stability test.

Figure S6. Lattice vibrational mode after photocatalytic stability test. Raman spectra peak of the F14-SnS_2_ sample after stability test. Our observation indicates that the peak positions of the film remained unchanged after the stability test.

Figure S7. Isotope experiments. a) Relative mass spectrum of the ^13^CO. b) Blank air measurement.

Figure S8. Normalized NAP-XPS signal versus relative binding energy of the grown thin films. a) C 1*s* and O 1*s* signals from the SnS_2_. b) C 1*s* and O 1*s* signals from the F14-SnS_2_. in this context, labels A to E represent various reaction conditions, including: (A) Ultra-high vacuum (UHV); (B) In a CO_2_ atmosphere in the dark; (C) In a CO_2_ atmosphere under light illumination; (D) In a CO_2_ + H_2_O atmosphere in the dark; and (E) In a CO_2_ + H_2_O atmosphere under light illumination. Relative Binding Energy: $\Delta BE=BE-{BE}_{C-C}$ for C 1*s* and $\Delta BE=BE-{BE}_{COx}$ for O 1*s*.

Figure S9. NAP-XPS spectra of the grown thin films. a) Sn 3*d*, S 2*p*, and F 1*s* signals from the F-SnS_2_. b) Sn *3d* and S *2p* signals from the SnS_2_. In this context, labels A to E represent various reaction conditions, including: (A) Ultra-high vacuum (UHV); (B) In a CO_2_ atmosphere in the dark; (C) In a CO_2_ atmosphere under light illumination; (D) In a CO_2_ + H_2_O atmosphere in the dark; and (E) In a CO_2_ + H_2_O atmosphere under light illumination.

Figure S10. VBM of NAP-XPS of the grown thin films under different environments. a) for SnS_2_**.** b) F14-SnS_2_.

Figure S11. Configurations of F-doped in different S sites of SnS_2_:F by DFT calculations. a–i) Side-view of the calculated configurations of the SnS_2_ (F-sub. @S), SnS_2_(2F-sub @S)-1, SnS_2_(2F-sub @S)-2, SnS_2_(2F-sub @S)-3, SnS_2_(2F-sub @S)-4, SnS_2_(2F-sub @S)-5, SnS_2_(2F-sub @S)-6, SnS_2_(2F-sub @S)-7 and SnS_2_(2F-sub @S)-8, respectively. Dark blue-, yellow- and cyan-filled circles stand for Sn, S, and F atoms, respectively.

Figure S12. Configurations of S_V_ in different S sites of S_V_ -SnS2 by DFT calculations. a–f) Top-view of the calculated configurations of the SnS_2_(S_V_), SnS_2_(2S_V_)-1, SnS_2_(2S_v_)-2, SnS_2_(2S_V_)-3, SnS_2_(2S_V_)-4 and SnS_2_(2S_V_)-5**,** respectively. Dark blue- and yellow-filled circles stand for Sn and S atoms, respectively. Dark orange circle shows S_V._

Figure S13. Configurations of S_V_ in different S sites of SnS_2_:F by DFT calculations. a–h) Top-view of the calculated configurations of the S_V_-SnS_2_:F-1, S_V_-SnS_2_:F-2, S_V_-SnS_2_:F-3, S_V_-SnS_2_:F-4, S_V_-SnS_2_:F-5, S_V_-SnS_2_:F-6, S_V_-SnS_2_:F-7 and S_V_-SnS_2_:F-8 respectively. Dark blue-, yellow- and cyan-filled circles stand for Sn, S, and F atoms, respectively. Dark orange circle shows S_V._

# Supplementary Tables

Table S1. Formation energy of F dopant in SnS_2_. The absolute formation energy of the calculated different configurations is shown in **Figure S1**.

|  | **lattice constant (Å)** | | | **Formation energy** |
| --- | --- | --- | --- | --- |
|  | $\boldsymbol{a}$ | $\boldsymbol{b}$ | $\boldsymbol{c}$ | $\boldsymbol{E}_{\boldsymbol{f}}$ **(eV)** |
| SnS_2_ | 14.727 | 14.727 | 11.939 | -- |
| SnS_2_ (F-sub. @S) | 14.719 | 14.719 | 11.947 | -2.0 |
| SnS_2_ (F-sub. @Sn) | 14.707 | 14.707 | 11.850 | 5.65 |
| SnS_2_ (F @ int.-1) | 14.738 | 14.738 | 12.055 | -0.27 |
| SnS_2_ (F @ int.-2) | 14.735 | 14.734 | 12.017 | -0.19 |
| SnS_2_ (F @ int.-3) | 14.738 | 14.738 | 12.050 | 0.31 |
| S_V_-SnS_2_ | 14.699 | 14.699 | 11.949 | 2.13 |
| Sn_V_-SnS_2_ | 14.727 | 14.727 | 11.751 | 6.40 |

Table S2. XPS fitted peak positions for SnS_2_ and F14-SnS_2_ films. Peak positions and FWHMs of the XPS fitted peak for Sn 3*d*_5/2_, S 2*p*_3/2_, and F 1*s* elements. The FWHMs are added in the parentheses.

| **Sample** | Sn 3*d*_5/2_ | S 2*p*_3/2_ | F 1*s* |
| --- | --- | --- | --- |
| **SnS_2_** | 486.311 (1.17) | 161.306  (1.09) | -  - |
| **F14-SnS_2_** | 486.37  (1.39) | 161.33  (1.45) | 689.26  (1.76) |

Table S3. Calculation details of $AQE$ and $IQE$. Overall $AQE$ of PC CO_2_ reduction over a catalyst under the Xe lamp.

| Light source | Solar simulator (AM1.5) |
| --- | --- |
| Photon flux (cm^-2^ s^-1^) | 2.43 × 10^17^ |
| CO-yield (µmol) | XX |
| Acetaldehyde-yield (µmol) | YY |
| Irradiation area (cm^2^) | 1 |
| Irradiation time (s) | 4 × 3600 |
| $\boldsymbol{AQE}_{\mathbf{overall}}$ (%) | ZZ in % |
| $\boldsymbol{IQE}$ (%) | ZZ in % |

The quantity of overall ${AQE}_{\mathrm{overall}}$is calculated by the following equation,

${AQE}_{\mathrm{overall}}=\frac{[\left( Ne\times\mu mol \mathrm{of}Acetaldehyde Yield \right)+(Ne\times\mu mol\mathrm{of}CO Yield )]\times6.02\times{10}^{23}}{Photon flux\times Irradiation area\times Irradiation time}\times100\%$,

$IQE =\frac{AQE}{\beta}$,

where $Ne$ and $\beta$ the number of reacted electrons and the percentage of absorbed light.

# Table S4. Photocatalytic CO_2_ reduction productivity comparison.

| No. | Year | Co-catalyst @ catalyst (mass, exposed area) | Light source  (Lamp)  (Power) | Product | Product rate | Quantum efficiency (%) | Selectivity towards the major product | Ref. |
| --- | --- | --- | --- | --- | --- | --- | --- | --- |
| 1 | This work | F14-SnS_2_ continuous thin film (1cm^2^) | solar simulator  (AM 1.5) | CO  CH_3_COH | 0.39 μmol cm^-2^ h^-1^  0.011 μmol cm^-2^ h^-1^ | $IQE$=0.52% $AQE$=0.06% | ~97% | This work |
| 2 | 2025 | ZnIn_2_S_4_/g-C_3_N_4_ |  | CO | 166.5 μmol g^-1^ h^-1^ | $AQE$=5.7%  (at 420 nm) | 100% | [1] |
| 3 | 2025 | Ov-Ni-Bi_2_WO_6_ powder | solar simulator  (AM 1.5) | CO | 13.37 μmol g^-1^ h^-1^ | $AQE$=0.013% | 100% | [2] |
| 4 | 2024 | S_V_-SnS_2_:P continuous thin film (1cm^2^) | solar simulator  (AM 1.5) | CH_4_  CH_3_COH | 0.0325 μmol cm^-2^ h^-1^  0.0031 μmol cm^-2^ h^-1^ | $AQE$=0.02% | 92% | [3] |
| 5 | 2024 | 1.5Fe-Bi_2_WO_6_ | solar simulator  (AM 1.5) | CO | 9.195 μmol g^-1^ h^-1^ | $AQE$=0.009%^a^ | 100% | [4] |
| 6 | 2022 | Amine-functionalized ultrathin nanoporous boron-doped g- C_3_N_4_ (AUNB/g-C_3_N_4_) | solar simulato  (AM 1.5) | CO | 5.49 μmol g^-1^ h^-1^ | $AQE$=0.004%^a^ | 100% | [5] |
| 7 | 2022 | ZnS/ZnIn_2_S_4_ | solar simulator  (AM 1.5) | CH_3_CHO  CH_3_OH | 61.272 μmol g^-1^ h^-1^  0.223 μmol g^-1^ h^-1^ | $AQE$=0.8% | 99.6% | [6] |
| 8 | 2022 | WSe­_2_ monolayers | solar simulator  (AM 1.5) | CH_4_  CH_3_COH | NA | $IQE$=0.23% | 90% | [7] |
| 9 | 2020 | g-C_3_N_4_/Pt/3DOM-TiO_2_@C catalysts | 300 W xenon lamp was chosen to simulate a solar external filter  (420 nm) | CO  CH_4_ | 1.47 μmol g^-1^ h^-1^  6.56 μmol g^-1^ h^-1^ | $AQE$=0.05% | 81.7% | [8] |
| 10 | 2020 | C–SnS_2_ thin film | solar simulator  (AM 1.5) | CH_4_  CH_3_CHO | 0.125 μmol cm^-2^ h^-1^  0.015 μmol cm^-2^ h^-1^ | $AQE$=0.07%^a^ | 85% | [9] |
| 11 | 2020 | Fe–based MOF | 300 W Xenon  400-780 nm  (0.4 W cm^-2^ | CO  CH_4_ | ~4 μmol g^-1^ h^-1^  ~50 μmol g^-1^ h^-1^ | $AQE$= 0.924% Overall | 98% | [10] |
| 12 | 2019 | Vs–CuIn_5_S_8_ | Xenon > 420 nm  (0.05 W cm^-2^) | CH_4_ | 8.7 μmol g^-1^ h^-1^ | $AQE$= 0.786% Overall | 100% | [11] |
| 13 | 2019 | SnS_2_/TiO_2_ | 300 W Xe lamp | CO  CH_4_ | ~2.5 μmol g^-1^ h^-1^  23 μmol g^-1^ h^-1^ | - | 90% | [12] |
| 14 | 2018 | Hierarchical treated rape pollen (TRP) | 300 W Xenon lamp  with cutoff filter 400 nm | CO  CH_4_ | 488.4 μmol g^-1^ h^-1^  8.4 μmol g^-1^ h^-1^ | $AQE$=6.7%  (at 420 nm) | 98.3% | [13] |
| 15 | 2018 | C–SnS_2_ powder | solar simulator  (AM 1.5) | CH_3_CHO | 13.98 μmol g^-1^ h^-1^ | $AQE$=0.7% | 100% | [14] |
| 16 | 2017 | Mildly oxidized SnS_2_ atomic layers | Xe lamp  300 W with a standard AM 1.5 filter and a 420 nm cutoff filter | CO | 12.28 μmol g^-1^ h^-1^ | - | 100% | [15] |
| 17 | 2017 | g-C3N4/SnS_2_ | 300 W Xe light  with cutoff filter 400 nm | CH_4_  CH_3_CHO | 0.2133 μmol g^-1^ h^-1^  0.7466 μmol g^-1^ h^-1^ | - | 77.8% | [16] |

^a^Calculated by authors.

Table S5. In-situ NAP-XPS fitted peak position for Sn 3*d*_5/2_, S 2*p*_3/2_, and F 1*s* elements of SnS_2_ and F-SnS_2_ samples. In this context, labels A to E represent various reaction conditions, including: (A) Ultra-high vacuum (UHV); (B) In a CO_2_ atmosphere in the dark; (C) In a CO_2_ atmosphere under light illumination; (D) In a CO_2_ + H_2_O atmosphere in the dark; and (E) In a CO_2_ + H_2_O atmosphere under light illumination. Peak positions and FWHMs of the X-ray photoelectron spectroscopy fitted peak for Sn 3*d*_5/2_, S 2*p*_3/2_, and F 1*s* elements. The FWHMs are added in the parentheses.

| Condition | SnS_2_ | | F-SnS_2_ | | |
| --- | --- | --- | --- | --- | --- |
|  | **Sn 3*d*_5/2_** | **S 2*p*_3/2_** | **Sn 3*d*_5/2_** | **S 2*p*_3/2_** | **F 1*s*** |
| A | 488.11  *(11.087)* | 163.11  *(1.04)* | 487.99  *(1.24)* | 162.97  *(1.05)* | 688.88  *(2.49)* |
| B | 488.22  (*1.11*) | 163.14  *(1.03)* | 488.19  *(1.25)* | 163.24  *(1.07)* | 689.09  *(2.22)* |
| C | 488.19  (*1.09*) | 163.21  *(1.03)* | 488.33  *(1.26)* | 163.38  *(1.08)* | 689.14  *(2.75)* |
| D | 488.38  (*1.13*) | 163.38  *(1.04)* | 488.46  *(1.26)* | 163.45  *(1.09)* | 689.46  *(3)* |
| E | 488.33  (*1.11*) | 163.36  *(1.02)* | 488.40  *(1.26)* | 163.37  *(1.08)* | 689.48  *(2.49)* |

Table S6. In-situ NAP-XPS fitted peak position for C 1*s*, and O 1*s* elements of SnS_2_ and F-SnS_2_ samples. In this context, labels A to E represent various reaction conditions, including: (A) Ultra-high vacuum (UHV); (B) In a CO_2_ atmosphere in the dark; (C) In a CO_2_ atmosphere under light illumination; (D) In a CO_2_ + H_2_O atmosphere in the dark; and (E) In a CO_2_ + H_2_O atmosphere under light illumination. Peak positions and FWHMs of the X-ray photoelectron spectroscopy fitted peak for C *1s*, and O *1s* elements. The FWHMs are added in the parentheses.

| Condition | SnS_2_ | | | | | | F-SnS_2_ | | | | | |
| --- | --- | --- | --- | --- | --- | --- | --- | --- | --- | --- | --- | --- |
|  | **C 1*s*** | | | | **O 1*s*** | | **C 1*s*** | | | | **O 1s** | |
|  | C-C | C-O | C=O | O-C=O | COx | O-Si | C-C | C-O | C=O | O-C=O | COx | O-Si |
| A | 286.21  *(1.47)* | 287.98  *(1.3)* | 289.31  (1.34) | 290.36  (1.3) | 534.02  (1.55) | 531.72  (1.55) | 286.11  (1.46) | 287.61  (1.6) | 289.06  (1.6) | 290.48  (1.25) | 533.94  (1.62) | 532.00  *(1.62)* |
| B | 286.27  (*1.44)* | 287.90  (1.44) | 289.07  (1.5) | 290.38  (1.55) | 534.25  (1.62) | 531.97  (1.62) | 286.19  (1.47) | 287.61  (1.69) | 289.00  (1.67) | 290.30  (1.33) | 534.08  (1.69) | 532.06  *(1.69)* |
| C | 286.29  *(1.43)* | 287.85  (1.42) | 289.09  (1.43) | 290.51  (1.44) | 534.29  (1.61) | 532.00  (1.61) | 286.38  (1.49) | 287.69  (1.72) | 289.00  (1.69) | 290.30  (1.52) | 534.36  (1.74) | 532.39  *(1.74)* |
| D | 286.47  *(1.42)* | 288.01  (1.55) | 289.27  (1.55) | 290.39  (1.55) | 534.46  (1.5) | 532.28  (1.58) | 286.51  (1.48) | 287.74  (1.69) | 289.00  (1.66) | 290.28  (1.45) | 534.51  (1.70) | 532.50  *(1.79)* |
| E | 286.47  *(1.40)* | 287.86  (1.55) | 289.275(1.55) | 290.67  (1.27) | 534.43  (1.56) | 532.26  (1.56) | 286.55  (1.48) | 287.83  (1.69) | 289.00  (1.69) | 290.26  (1.59) | 534.48  (1.70) | 532.50  *(1.79)* |

Table S7. Desorption energy of CO. The desorption energy of CO on SnS_2_(S active site), Sv-SnS_2_ (Sn active site), Sv-SnS_2_:F (F active site), and Sv-SnS_2_:F (F active site) is calculated with respect to a reference energy of CO_2_ + *.

| Co desorption | SnS_2_  (S active site) | S_V_-SnS_2_  (Sn active site) | S_V_-SnS_2_:F  (F active site) | S_V_-SnS_2_:F  (S active site) | S_V_-SnS_2_:F  (Sn active site) |
| --- | --- | --- | --- | --- | --- |
| CO* | 0.66 | 0.17 | 0.71 | 1.22 | -0.11 |
| CO + * | 0.72 | 0.72 | 0.97 | 1.45 | 0.17 |
| COH* | 1.03 | 0.42 | 0.95 | 1.24 | 0.45 |
| CO Desorption energy (eV) | 0.06 | 0.55 | 0.26 | 0.23 | 0.28 |
| COH* from CO* | 0.37 | 0.25 | 0.24 | 0.02 | 0.56 |

Reference energy is from CO_2_ + *

Table S8. Relative formation energy of F in different S sites of SnS_2_:F. The formation energy of the calculated different configurations of two F ion by fixing one F in one place and varying the other one in different S sites is shown in **Figure S11.**

| System | Relative formation energy (eV) | |
| --- | --- | --- |
| SnS_2_(F-sub @S) | -2.00 |  |
| SnS_2_(2F-sub @S)-1 | -2.60 |  |
| SnS_2_(2F-sub @S)-2 | -2.07 |  |
| SnS_2_(2F-sub @S)-3 | -1.93 |  |
| SnS_2_(2F-sub @S)-4 | -2.03 |  |
| SnS_2_(2F-sub @S)-5 | -2.25 |  |
| SnS_2_(2F-sub @S)-6 | -2.13 |  |
| SnS_2_(2F-sub @S)-7 | -2.09 |  |
| SnS_2_(2F-sub @S)-8 | -2.00 |  |

Table S9. Relative formation energy of S_V_ in different S sites of S_V_-SnS2. The formation energy of the calculated different configurations of two Sulfur vacancies (S_V_) by fixing one Sulfur vacancy (S_V_) in one place and varying the other one in different S sites is shown in **Figure S12.**

| System | Relative formation energy (eV) |
| --- | --- |
| SnS_2_ (S_V_) | 1.25 |
| SnS_2_(2S_V_)-1 | 0.48 |
| SnS_2_(2S_V_)-2 | 1.16 |
| SnS_2_(2S_V_)-3 | 1.61 |
| SnS_2_(2S_V_)-4 | 1.44 |
| SnS_2_(2S_V_)-5 | 1.9 |

Table S10. Relative formation energy of S_V_ in different S sites of SnS_2_:F. The formation energy of the calculated different configurations of S_V_ by fixing one F in one place and varying one S_V_ in different S sites is shown in **Figure S13.**

| System | Relative formation energy (eV) |
| --- | --- |
| S_V_-SnS_2_:F-1 | 2.13 |
| S_V_-SnS_2_:F-1 | 1.68 |
| S_V_-SnS_2_:F-2 | 1.65 |
| S_V_-SnS_2_:F-3 | 1.67 |
| S_V_-SnS_2_:F-4 | 1.04 |
| S_V_-SnS_2_:F-5 | 1.33 |
| S_V_-SnS_2_:F-6 | 1.53 |
| S_V_-SnS_2_:F-7 | 1.66 |

# Supplementary Notes

# Note S1. Growth of F-implanted SnS_2_ thin film

**Simulation and implementation.** The implantation depth at different energies is simulated using the stopping and range of ions in matter (SRIM) software package. As shown in **Figure N1**, results show that an ion energy of 5 keV is suitable for a 20-nm-thick SnS_2_ thin film. Therefore, a series of F ion implantations were introduced to the as-grown SnS_2_ thin-film sample with different ion doses 1×10^13^_,_ 1×10^14,^ and 1×10^15^ ions cm^-2^, accomplished using a ULVAC IMX3500 ion-implanter equipped with multiple ion sources.

Figure N1. SRIM simulation. The ion implantation depth profile at the same F ion dose and different ion energies.

# Supplementary References

[1] H.A.E. Omr, R. Putikam, M.K. Hussien, A. Sabbah, T.Y. Lin, K.H. Chen, L.C. Chen, H.L. Wu, S.P. Feng, M.C. Lin, H. Lee, Unveiling the role of Zn−N1S_3_ sites in atomic-precision ZnIn_2_S_4_/g-C_3_N_4_ heterostructure for highly efficient CO_2_-to-CO conversion, Chem. Eng. J. 526 (2025). https://doi.org/10.1016/j.cej.2025.170766.

[2] N.Q. Thang, A. Sabbah, R. Putikam, C.Y. Huang, T.Y. Lin, M.K. Hussien, H.L. Wu, M.C. Lin, C.H. Lee, K.H. Chen, L.C. Chen, Regulating COOH Intermediate via Rationally Constructed Surface-Active Sites of Bi_2_WO_6_ for Solar-Driven CO2-to-CO Production, Adv. Funct. Mater. 2423751 (2025) 1–13. https://doi.org/10.1002/adfm.202423751.

[3] T.T. Mamo, M. Qorbani, A.G. Hailemariam, R. Putikam, C.M. Chu, T.R. Ko, A. Sabbah, C.Y. Huang, S. Kholimatussadiah, T. Billo, M.K. Hussien, S.Y. Chang, M.C. Lin, W.Y. Woon, H.L. Wu, K.T. Wong, L.C. Chen, K.H. Chen, Enhanced CO_2_ photoreduction to CH_4_ via *COOH and *CHO intermediates stabilization by synergistic effect of implanted P and S vacancy in thin-film SnS2, Nano Energy 128 (2024) 109863. https://doi.org/10.1016/j.nanoen.2024.109863.

[4] N.Q. Thang, A. Sabbah, C.Y. Huang, N.H. Phuong, T.Y. Lin, M.K. Hussien, H.L. Wu, C.I. Wu, N.N.T. Pham, P. Van Viet, C.H. Lee, L.C. Chen, K.H. Chen, Tailoring atomically dispersed Fe-induced oxygen vacancies for highly efficient gas-phase photocatalytic CO_2_ reduction and NO removal with diminished noxious byproducts, J. Mater. Chem. A (2024). https://doi.org/10.1039/d4ta05778c.

[5] M. Kamal Hussien, A. Sabbah, M. Qorbani, M. Hammad Elsayed, P. Raghunath, T.Y. Lin, S. Quadir, H.Y. Wang, H.L. Wu, D.L.M. Tzou, M.C. Lin, P.W. Chung, H.H. Chou, L.C. Chen, K.H. Chen, Metal-free four-in-one modification of g-C_3_N_4_ for superior photocatalytic CO_2_ reduction and H_2_ evolution, Chem. Eng. J. 430 (2022) 132853. https://doi.org/10.1016/j.cej.2021.132853.

[6] A. Sabbah, I. Shown, M. Qorbani, F.Y. Fu, T.Y. Lin, H.L. Wu, P.W. Chung, C.I. Wu, S.R.M. Santiago, J.L. Shen, K.H. Chen, L.C. Chen, Boosting photocatalytic CO2 reduction in a ZnS/ZnIn_2_S_4_ heterostructure through strain-induced direct Z-scheme and a mechanistic study of molecular CO_2_ interaction thereon, Nano Energy 93 (2022) 106809. https://doi.org/10.1016/j.nanoen.2021.106809.

[7] M. Qorbani, A. Sabbah, Y.R. Lai, S. Kholimatussadiah, S. Quadir, C.Y. Huang, I. Shown, Y.F. Huang, M. Hayashi, K.H. Chen, L.C. Chen, Atomistic insights into highly active reconstructed edges of monolayer 2H-WSe_2_ photocatalyst, Nat. Commun. 13 (2022) 1–8. https://doi.org/10.1038/s41467-022-28926-0.

[8] C. Wang, X. Liu, W. He, Y. Zhao, Y. Wei, J. Xiong, J. Liu, J. Li, W. Song, X. Zhang, Z. Zhao, All-solid-state Z-scheme photocatalysts of g-C_3_N_4_/Pt/macroporous-(TiO_2_@carbon) for selective boosting visible-light-driven conversion of CO_2_ to CH_4_, J. Catal. 389 (2020) 440–449. https://doi.org/10.1016/j.jcat.2020.06.026.

[9] T. Billo, I. Shown, T. Amerta, A. Sabbah, F. Fu, C. Chu, W. Woon, R. Chen, H. Lee, K. Chen, L. Chen, Nano Energy A mechanistic study of molecular CO_2_ interaction and adsorption on carbon implanted SnS_2_ thin film for photocatalytic CO_2_ reduction activity, Nano Energy 72 (2020) 104717. https://doi.org/10.1016/j.nanoen.2020.104717.

[10] X.Y. Dao, J.H. Guo, X.Y. Zhang, S.Q. Wang, X.M. Cheng, W.Y. Sun, Structure-dependent iron-based metal-organic frameworks for selective CO_2_-to-CH_4_ photocatalytic reduction, J. Mater. Chem. A 8 (2020) 25850–25856. https://doi.org/10.1039/d0ta10278d.

[11] X. Li, Y. Sun, J. Xu, Y. Shao, J. Wu, X. Xu, Y. Pan, H. Ju, J. Zhu, Y. Xie, Selective visible-light-driven photocatalytic CO_2_ reduction to CH_4_ mediated by atomically thin CuIn_5_S_8_ layers, Nat. Energy 4 (2019) 690–699. https://doi.org/10.1038/s41560-019-0431-1.

[12] H. She, H. Zhou, L. Li, Z. Zhao, M. Jiang, J. Huang, L. Wang, Q. Wang, Construction of a Two-Dimensional Composite Derived from TiO_2_ and SnS_2_ for Enhanced Photocatalytic Reduction of CO_2_ into CH_4_, ACS Sustain. Chem. Eng. 7 (2019) 650–659. https://doi.org/10.1021/acssuschemeng.8b04250.

[13] Z. Jiang, H. Sun, T. Wang, B. Wang, W. Wei, H. Li, S. Yuan, T. An, H. Zhao, J. Yu, P.K. Wong, Nature-based catalyst for visible-light-driven photocatalytic CO_2_ reduction, Energy Environ. Sci. 11 (2018) 2382–2389. https://doi.org/10.1039/c8ee01781f.

[14] I. Shown, S. Samireddi, Y.C. Chang, R. Putikam, P.H. Chang, A. Sabbah, F.Y. Fu, W.F. Chen, C.I. Wu, T.Y. Yu, P.W. Chung, M.C. Lin, L.C. Chen, K.H. Chen, Carbon-doped SnS2 nanostructure as a high-efficiency solar fuel catalyst under visible light, Nat. Commun. 9 (2018). https://doi.org/10.1038/s41467-017-02547-4.

[15] X. Jiao, X. Li, X. Jin, Y. Sun, J. Xu, L. Liang, H. Ju, J. Zhu, Y. Pan, W. Yan, Y. Lin, Y. Xie, Partially Oxidized SnS_2_ Atomic Layers Achieving Efficient Visible-Light-Driven CO_2_ Reduction, J. Am. Chem. Soc. 139 (2017) 18044–18051. https://doi.org/10.1021/jacs.7b10287.

[16] T. Di, B. Zhu, B. Cheng, J. Yu, J. Xu, A direct Z-scheme g-C_3_N_4_/SnS_2_ photocatalyst with superior visible-light CO_2_ reduction performance, J. Catal. 352 (2017) 532–541. https://doi.org/10.1016/j.jcat.2017.06.006.
